# Corydaline Inhibits Multiple Cytochrome P450 and UDP-Glucuronosyltransferase Enzyme Activities in Human Liver Microsomes

**DOI:** 10.3390/molecules16086591

**Published:** 2011-08-05

**Authors:** Hye Young Ji, Kwang Hyeon Liu, Hyeri Lee, Sae Rom Im, Hyun Joo Shim, Miwon Son, Hye Suk Lee

**Affiliations:** 1 Drug Metabolism and Bioanalysis Laboratory, College of Pharmacy, The Catholic University of Korea, Bucheon 420-743, Korea; 2 College of Pharmacy and Research Institute of Pharmaceutical Sciences, Kyungpook National University, Daegu 702-701, Korea; 3 Department of Agricultural Biotechnology, Seoul National University, Seoul 151-742, Korea; 4 Research Center, Dong-A Pharmaceutical Co., Yongin 446-905, Korea

**Keywords:** corydaline, cytochrome P450 inhibition, UDP-glucuronosyltransferase inhibition, human liver microsomes, drug-drug interaction

## Abstract

Corydaline is a bioactive alkaloid with various antiacetylcholinesterase, antiallergic, and antinociceptive activities found in the medicinal herb Corydalis Tubers. The inhibitory potential of corydaline on the activities of seven major human cytochrome P450 and four UDP-glucuronosyltransferase enzymes in human liver microsomes was investigated using LC-tandem MS. Corydaline was found to inhibit CYP2C19-catalyzed *S*-mephenytoin-4’-hydroxylatoin and CYP2C9-catalyzed diclofenac 4-hydroxylation, with *K*_i_ values of 1.7 and 7.0 μM, respectively. Corydaline also demonstrated moderate inhibition of UGT1A1-mediated 17β-estradiol 3-glucuronidation and UGT1A9-mediated propofol glucuronidation with *K*_i_ values of 57.6 and 37.3 μM, respectively. In the presence of corydaline, CYP3A-mediated midazolam hydroxylation showed a decrease with increasing preincubation time in a dose-dependent manner with *K*_i_ values of 30.0 μM. These *in vitro* results suggest that corydaline should be evaluated for potential pharmacokinetic drug interactions *in vivo* due to potent inhibition of CYP2C19 and CYP2C9.

## 1. Introduction

**Figure 1:**
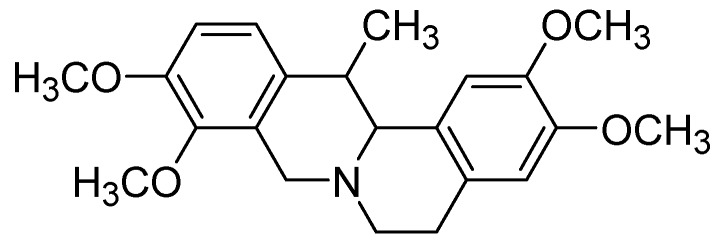
Chemical structure of corydaline.

Corydalis tubers, the roots of *Corydalis yanhusuo* W.T. Wang, have long been used as an herbal drug for their analgesic and anti-ulcer effects [1,2,3]. Corydaline, 2,3,9,10-tetramethoxy-13-methyl-5,8,13,13a-tetrahydro-6H-isoquino[3,2-a]isoquinoline (Figure 1), is isolated from Corydalis tubers and has been demonstrated to show antiacetylcholinesterase [4,5,6] and antibutylcholinesterase activity [4], antiallergic activity [7], and antinociceptive activity [8]. Corydaline also promotes gastric emptying and facilitates gastric accommodation [9].

Use of herbal medicine and botanical drugs to prevent common illness is on the rise among the global population [10]. Because botanical drugs share the same metabolic and transport proteins, including cytochrome P450 (CYP) enzymes, UDP-glucuronosyltransferase (UGT) enzymes, and drug transporters, such as P-glycoprotein, with commonly used drugs, the potential for herb-drug interactions is substantial [11]. Several medicinal herbs, including garlic (*Allium sativum*), ginkgo (*Ginko biloba*), ginseng (*Panax ginseng*), milk thistle (*Silybum marianum*), licorice (*Glycyrrhiza glabra*), and St. John’s wort (*Hypericum perforatum*), have been reported to cause such herb-drug interactions [12,13,14]. Therefore, interactions among therapeutic drugs, as well as interactions of drugs with food and herbal medications, have attracted considerable attention [15].

Modulation of drug-metabolizing enzymes, such as CYP and UGT enzymes, is one of the important mechanisms of drug interaction. Among the numerous CYP and UGT enzymes identified to date, seven human hepatic CYP enzymes (CYPs 1A2, 2A6, 2C8, 2C9, 2C19, 2D6, and 3A) and four UGT enzymes (UGTs 1A1, 1A4, 1A9, and 2B7) have been shown to a play important role in drug metabolism [16,17]. Studies have shown that bergamottin, a major furanocoumarin found in grapefruit juice, is reported to increase the blood concentration of drugs through inhibition of hepatic CYP3A activity, thereby enhancing the toxicity of drugs such as atorvastatin, felodipine, quinidine, sildenafil, and verapamil [18].

To the best of our knowledge, no previous study has reported on the effect of corydaline, a pharmacologically active isoquinoline alkaloid isolated from Corydalis tubers, on human CYP and UGT enzymes. In this study, the effects of corydaline on activities of seven major human CYP and four major human UGTs (1A1, 1A4, 1A9, and 2B7), were examined using pooled human liver microsomes in order to evaluate the possibility of corydaline-drug interactions.

## 2. Results and Discussion

**Figure 2:**
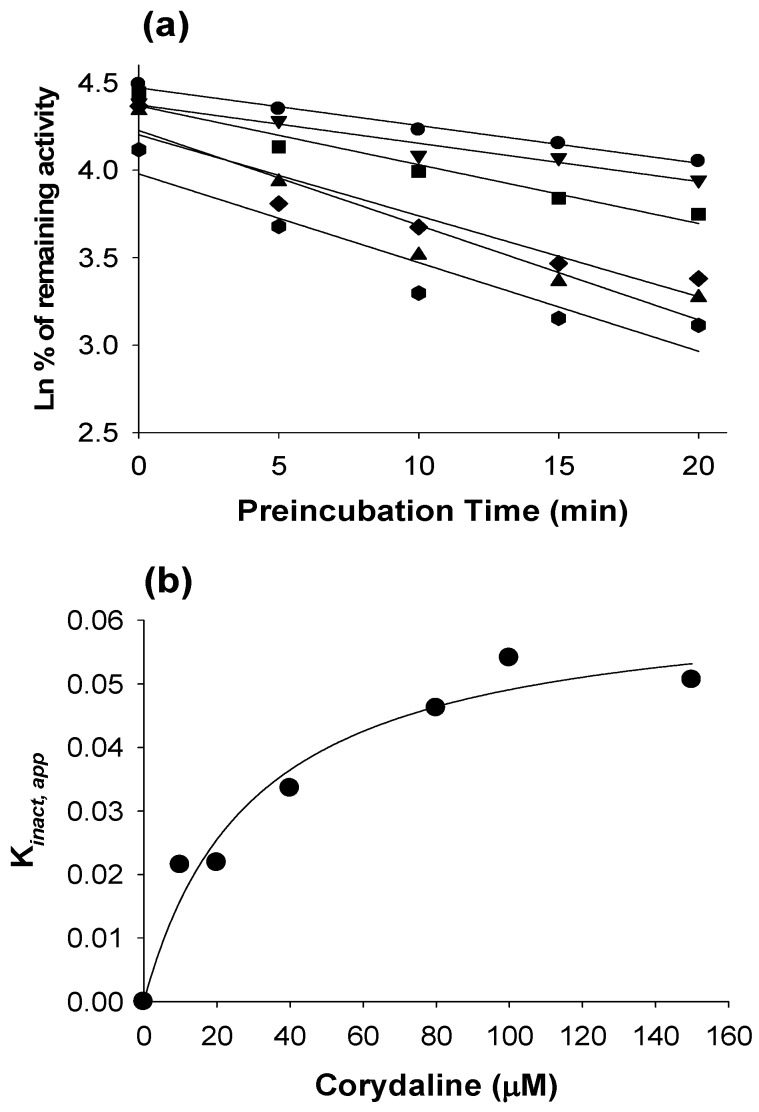
Kinetics of inactivation of microsomal formation of 1’-hydroxymidazolam from midazolam by different concentrations of corydaline; ⬤, 10 μM; ▼, 20 μM; ■, 40 μM; ◆, 80 μM; ▲, 100 μM; and ⬢, 150 μM (**a**); Relationship between *k*_obs_ and corydaline concentration, used to estimate values of *k*_inact_ and *K*_i_ (**b**).

The inhibitory effects of corydaline toward seven major human CYP isoforms and four major human UGT isoforms were evaluated using cocktail CYP probe substrates and each UGT probe substrate in human liver microsomes, respectively. Corydaline induced moderate inhibition of CYP2C19-mediated *S*-mephenytoin 4-hydroxylation and CYP2C9-mediated diclofenac 4-hydroxylation with IC_50_ values of 11.7 and 26.2 μM, respectively. Corydaline also induced weak inhibition of CYP2D6-catalyzed bufuralol 1’-hydroxylation with an IC_50_ value of 64.5 μM. This is not an unusual finding, given that berberine and palmatine, isoquinoline alkaloids like corydaline, also showed an inhibitory effect on CYP2D6 activity (with IC_50_ values of 49.4 and 92.6 μM, respectively) [19,20]. Corydaline at 200 μM showed negligible inhibition of CYP1A2-mediated phenacetin *O*-deethylation, CYP2A6-mediated coumarin 7-hydroxylation, CYP2C8-mediated amodiaquine *N*-deethylation, and CYP3A-mediated midazolam 1’-hydroxylation. The inhibitory potencies of corydaline were not significantly affected after a 30-min period of pre-incubation with human liver microsomes in the presence of NADPH, excluding CYP3A activity (Table 1), indicating that corydaline is a mechanism based inhibitor of CYP3A. To determine whether midazolam metabolism is inhibited by corydaline, we performed time- and concentration-dependent inactivation assays in human liver microsomes. In the presence of corydaline, CYP3A-mediated midazolam hydroxylation was decreased with increasing preincubation time in a dose-dependent manner (Figure 2a). Values for *k*_obs_ were plotted against corydaline concentration and used for estimation of maximal rates (*k*_inact_) and *K*_i_ values in human liver microsomes (Figure 2b). Estimated maximal rates (*k*_inact_) and apparent *K*_i_ values were 0.064 min^−1^ and 30.0 μM in human liver microsomes, respectively.

**Figure 3:**
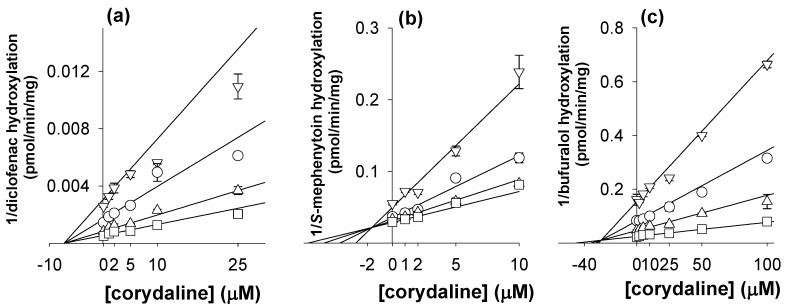
Representative Dixon plots for inhibitory effects of corydaline on (**a**) CYP2C9-catalyzed diclofenac 4-hydroxylation; (**b**) CYP2C19-catalyzed [*S*]-mephenytoin 4-hydroxylation; and (**c**) CYP2D6-catalyzed bufuralol hydroxylation in pooled human liver microsomes (H161). Each symbol represents the substrate concentration: (a) diclofenac, 1 μM (▽), 2 μM (◯), 5 μM (△), 10 μM (☐); (b) *S*-mephenytoin, 10 μM (▽), 20 μM (◯), 30 μM (△), 40 μM (☐); (c) bufuralol, 0.5 μM (▽), 1.0 μM (◯), 2.0 μM (△), 5.0 μM (☐). Each data point represents the mean of triplicate experiments.

In an inhibition study, the apparent *K*_i_ value is a better parameter for defining the interaction of the inhibitor with a particular enzyme. *K*_i_ values and inhibition types (competitive, noncompetitive, uncompetitive, or mixed) for corydaline were determined using Lineweaver plots, Dixon plots, and secondary reciprocal plots; the results are summarized in Table 1. Corydaline showed noncompetitive inhibition for CYP2C9-catalyzed diclofenac 4-hydroxylation (*K*_i_, 7.1 μM) and competitive inhibition for CYP2C19-catalyzed *S*-mephenytoin 4’-hydroxylation (*K*_i_, 1.7 μM) (Figure 3 and Table 1). Corydaline competitively inhibited CYP2D6-catalyzed bufuralol 1’-hydroxylation with a *K*_i_ value of 27.3 μM.

**Figure 4:**
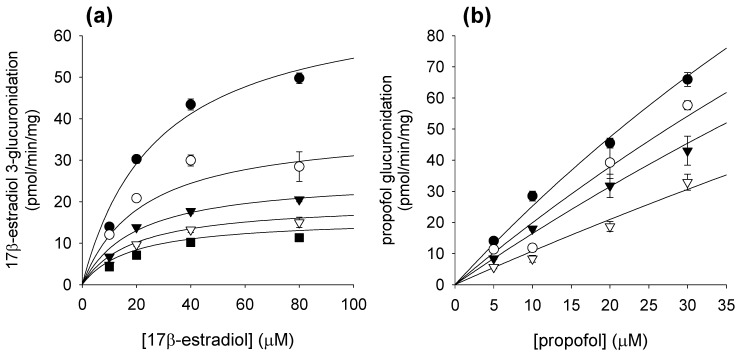
Effects of corydaline on rates of (**a**) UGT1A1-catalyzed 17β-estradiol 3-glucuronidation and (**b**) UGT1A9-catalyzed propofol glucuronidation in pooled human liver microsomes (H161). Lines represent the functions determined by nonlinear regression based on mixed competitive-noncompetitive equation. Each symbol represents the corydaline concentration: (a) 0 μM (⬤), 30 μM (◯), 60 μM (▼), 90 μM (▽), 120 μM (■); (b) 0 μM (⬤), 10 μM (◯), 20 μM (▼), 50 μM (▽). Each data point represents the mean of triplicate experiments.

We also evaluated the inhibitory potential of corydaline on UGT isoform activities. Corydaline inhibited UGT1A9-mediated propofol glucuronidation and UGT1A1-mediated 17β-estradiol 3-glucuronidation with IC_50_ values of 39.4 and 137.1 μM, respectively. UGT1A4-mediated trifluoperazine *N*-glucuronidation and UGT2B7-mediated azidothymidine glucuronidation were not inhibited by treatment with corydaline (Table 2). Corydaline inhibited UGT1A1-catalyzed 17β-estradiol 3-glucuronidation and UGT1A9-dependent propofol glucuronidation with *K*_i_ values of 57.6 and 37.3 μM, respectively (Figure 4 and Table 2). However, corydaline was not found to be a potent inhibitor of UGT1A1 and UGT1A9, as compared with inhibition produced by ritonavir (IC_50_ = 1.7 μM) and niflumic acid (*K*_i_ = 0.1~0.4 μM), an inhibitor of UGT1A1 [21] and UGT1A9 [22], respectively.

Corydaline was found to be a potent competitive inhibitor of CYP2C19 with *K*_i_ values of 1.7 μM, indicating that corydaline may be used carefully with drugs metabolized by CYP2C19, such as proton pump inhibitors (lansoprazole and omeprazole), antiepileptics (diazepam and phenytoin), and antidepressants (amitriptyline and imipramine) in order to avoid drug interactions. Some natural compounds, including anthocyanidin [23], beauvericin [24], eupatilin [25], and ursolic acid [26], have demonstrated strong inhibition of CYP2C19. Corydaline was also shown to be an inhibitor of CYP2C9 with *K*_i_ values of 7.1 μM, suggesting that corydaline should be used carefully with drugs metabolized by CYP2C9, such as celecoxib, diclofenac, glyburide, losartan, tolbutamide, torasemide, and *S*-warfarin in order to avoid drug interactions [27]. However, corydaline was not found to be a potent inhibitor of CYP2C9, as compared with the inhibition produced by sulfaphenazole (IC_50_ = 0.8 μM), a selective inhibitor of CYP2C9 [28]. Herbal preparations containing corydaline may also affect CYP2C19 and CYP2C9 activity. These results suggest the possibility that high amounts of corydaline might alter the bioavailability of drugs that are metabolized by CYP2C19 or CYP2C9, although *in vitro* inhibition of CYP2C19 and CYP2C9 activities don’t necessarily translate into *in vivo* drug interactions. Up to now, there was no available data on the pharmacokinetics of corydaline in human which is indispensable for the prediction of drug-drug interaction potential of corydaline. According to the recent report of Zhang *et al.* [29], corydaline contents in various batches of Yuanhu Zhitong tablets, a traditional Chinese medicine used for the treatment of nerve pain and stomach pain, didn’t exceed 1 mg/g of original preparations, which may cause very low blood concentrations (<0.5 μM), even when an entire oral dose reaches the systemic circulation. Therefore, the probability of clinically important drug interactions by corydaline might be negligible based on available data.

## 3. Experimental

### 3.1. Materials and Reagents

Corydaline (>98%) was obtained from Wako Pure Chemical Industries, Ltd. (Osaka, Japan). Acetaminophen, alamethicin, coumarin, diclofenac, 17β-estradiol, 17β-estradiol 3-glucuronide, glucose-6-phosphate, glucose-6-phosphate dehydrogenase, 7-hydroxycoumarin, midazolam, β-nicotinamide adenine dinucleotide phosphate (NADP), NADP reduced form (NADPH), phenacetin, propofol, trifluoperazine, and uridine-5-diphosphoglucuronic acid trisoduim salt (UDPGA) were purchased from Sigma Chemical Co. (St. Louis, MO, USA). Pooled human liver microsomes (H161), ^13^C_2_,^15^N-acetaminophen, bufuralol, *N*-desethylamodiaquine, 1’-hydroxybufuralol, d_9_-1-hydroxybufuralol maleate, 4-hydroxydiclofenac, ^13^C_6_-4-hydroxydiclofenac, 4-hydroxymephenytoin, d_3_-4-hydroxy-mephenytoin, 1’-hydroxymidazolam, and *S*-mephenytoin were obtained from BD Gentest Co. (Woburn, MA, USA). Azidothymidine, azidothymidine glucuronide, and propofol glucuronide were obtained from Toronto Research Center (Toronto, ON, Canada). Acetonitrile and methanol (HPLC grade) were obtained from Burdick & Jackson Inc. (Muskegon, MI, USA) and the other chemicals were of the highest quality available.

### 3.2. Inhibitory Effects of Corydaline on Seven Major CYP Activities in Human Liver Microsomes

Inhibitory potencies (IC_50_ values) of corydaline were determined using CYP assays in the presence and absence of corydaline (final concentrations of 1–200 μM with an acetonitrile concentration less than 0.5% v/v) using pooled human liver microsomes. Phenacetin *O*-deethylase, coumarin 7-hydroxylase, amodiaquine *N*-deethylase, diclofenac 4-hydroxylase, *S*-mephenytoin 4-hydroxylase, bufuralol hydroxylase, and midazolam 1’-hydroxylase activities were determined as probe activities for CYP1A2, CYP2A6, CYP2C8, CYP2C9, CYP2C19, CYP2D6, and CYP3A4, respectively, using cocktail incubation and liquid chromatography-tandem mass spectrometry (LC/MS/MS) [25]. The incubation mixtures were prepared in a total volume of 200 μL, as follows: pooled human liver microsomes (0.25 mg/mL), 1.3 mM NADPH, 3.3 mM MgCl_2_, 50 mM potassium phosphate buffer (pH 7.4), and a cocktail of probe substrates and various concentrations of corydaline (1−200 μM). The substrates were used at concentrations approximately equal to their respective *K*_m_ values: 50 μM phenacetin, 2.5 μM coumarin, 2.5 μM amodiaquine, 10 μM diclofenac, 100 μM *S*-mephenytoin, 5 μM bufuralol, and 2.5 μM midazolam. After a 3-min pre-incubation period at 37 °C, the reactions were initiated by addition of an NADP generating system and incubated for 20 min at 37 °C in a shaking water bath. After incubation, the reaction was stopped by placement of the tubes on ice and addition of 100 μL of ice-cold methanol containing internal standards (^13^C_2_,^15^N-acetaminophen for acetaminophen and *N*-desethylamodiaquine, d_5_-7-hydroxycoumarin for 7-hydroxycoumarin, ^13^C_6_-4-hydroxydiclofenac for 4-hydroxydiclofenac, d_3_-4-hydroxy-mephenytoin for 4-hydroxymephenytoin and hydroxymidazolam, d_9_-1-hydroxybufuralol for 1-hydroxybufuralol). The incubation mixtures were then centrifuged at 13,000 × g for 5 min. All incubations were performed in triplicate and the mean values were used. For evaluation of NADPH-dependent mechanism-based inhibition of CYP activities, various concentrations of corydaline (1−200 μM) were pre-incubated for 30 min with human liver microsomes in the presence of NADPH. The reaction was started by addition of a cocktail of CYP probe substrates.

All 7 metabolites produced from the cocktail of CYP isoform-specific substrates were simultaneously determined by LC/MS/MS [25]. The system consisted of a tandem quadrupole mass spectrometer (TSQ Quantum Access, ThermoFisher Scientific, San Jose, CA, USA) coupled with a Nanospace SI-2 LC system. Separation was performed on an Atlantis dC_18_ column (5 μm, 2.1 mm i.d. ×100 mm, Waters, Milford, MA, USA) using the gradient elution of a mixture of 5% methanol in 0.1% formic acid (mobile phase A) and 95% methanol in 0.1% formic acid (mobile phase B) at a flow rate of 0.25 mL/min: 10% mobile phase B for 1 min, 10% to 95% mobile phase B for 1 min, 95% mobile phase B for 5 min. The column and autosampler temperatures were 50 and 6 °C, respectively. After 1.5 min, the LC eluent was diverted from waste to the mass spectrometer fitted with the electrospray ionization (ESI) source and operated in positive ion mode. ESI source settings for ionization of the metabolites were as follows: electrospray voltage, 5.0 kV; vaporizer temperature, 420 °C; capillary temperature 360 °C; sheath gas pressure, 35 psi; auxiliary gas pressure, 10 psi. Quantification was performed by selected reaction monitoring (SRM) of the [M+H]^+^ ion and the related product ion for each metabolite. SRM transitions for the metabolites and internal standard are summarized in Table 3. Analytical data were processed using Xcalibur^®^ software (Thermo Fisher Scientific).

### 3.3. Inhibitory Effects of Corydaline on Four UGT Activities in Human Liver Microsomes

The inhibitory potencies (IC_50_ values) of corydaline were also determined with UGT assays in the presence and absence of corydaline (final concentrations of 1–1,000 μM with acetonitrile concentration less than 0.5% v/v) using pooled human liver microsomes. 17β-Estradiol 3-glucuronidation, trifluoperazine glucuronidation, propofol glucuronidation, and azidothymidine glucuronidation activities were determined as probe activities for UGT1A1, UGT1A4, UGT1A9, and UGT2B7, respectively, using LC/MS/MS. Incubation mixtures were prepared in a total volume of 100 μL, as follows: Pooled human liver microsomes (0.2 mg/mL for 17β-estradiol, trifluoperazine and azidothymidine; 0.1 mg/mL for propofol), 2 mM UDPGA, 25 μg/mL alamethicin, 10 mM MgCl_2_, 50 mM tris buffer (pH 7.4), UGT-isoform specific probe substrate (20 μM 17β-estradiol for UGT1A1, 5 μM trifluoperazine for UGT1A4, 10 μM propofol for UGT1A9, or 100 μM azidothymidine for UGT2B7), and various concentrations of corydaline (1−1000 μM). Reactions were initiated by addition of UDPGA, and incubations were carried out at 37 °C in a shaking water bath for 30 min. Reactions were terminated by addition of ice-cold methanol (100 μL) containing an internal standard (100 ng/mL ezetimibe for 17β-estradiol 3-glucuronide and propofol glucuronide; 30 ng/mL meloxicam for trifluoperazine glucuronide and azidothymidine glucuronide). The incubation mixtures were centrifuged at 13,000 × g for 5 min, followed by dilution of the supernatant (40 μL) with water (60 μL). Aliquots (5 μL) were then injected onto the LC/MS/MS system. All incubations were performed in triplicate and the mean values were used.

Glucuronides produced from UGT isoform-specific substrates were respectively determined by LC/MS/MS. Separation was performed on the Atlantis dC_18_ column using the gradient elution of a mixture of 5% methanol in 0.1% formic acid (mobile phase A) and 95% methanol in 0.1% formic acid (mobile phase B) at a flow rate of 0.25 mL/min: 10% mobile phase B for 2 min, 10% to 95% mobile phase B for 4 min. The column and autosampler temperatures were 50 and 6 °C, respectively. After 3.0 min, the LC eluent was diverted from waste to the mass spectrometer fitted with an ESI source. ESI source settings for ionization of trifluoperazine glucuronide and azidothymidine glucuronide in positive ion mode were as follows: Electrospray voltage, 5.0 kV; vaporizer temperature, 420 °C; capillary temperature 360 °C; sheath gas pressure, 35 psi; auxiliary gas pressure, 10 psi. ESI source settings for ionization of 17β-estradiol 3-glucuronide and propofol glucuronide in negative ion mode were as follows: Electrospray voltage, −4.0 kV; vaporizer temperature, 420 °C; capillary temperature 360 °C; sheath gas pressure, 35 psi; auxiliary gas pressure, 10 psi. Quantification was performed by SRM of the [M+H]^+^ ion for trifluoperazine glucuronide and azidothymidine glucuronide or [M−H]^−^ for 17β-estradiol 3-glucuronide and propofol glucuronide and the related product ion for each metabolite. SRM transitions for the metabolites and internal standard are summarized in Table 3.

### 3.4. Kinetic Analysis

For determination of *K*_i_ values for CYP2C9, CYP2C19, and CYP2D6, human liver microsomes (0.1 mg/mL) were incubated with various concentrations of substrates (1−10 μM diclofenac for CYP2C9, 10−40 μM *S*-mephenytoin for CYP2C19, and 0.5−5 μM bufuralol for CYP2D6), 1 mM NADPH, 10 mM MgCl_2_, and various concentrations of corydaline in 50 mM potassium phosphate buffer (pH 7.4) in a total incubation volume of 200 μL. Reactions were initiated by addition of NADPH at 37 °C and stopped after 15 min by placement of the incubation tubes on ice and addition of ice-cold methanol (100 μL) containing an internal standard (2.0 μg/mL ^13^C_6_-4-hydroxydiclofenac for diclofenac, 0.5 μg/mL d_3_-4-hydroxymephenytoin for mephenytoin, or 0.5 μg/mL d_9_-1-hydroxy-bufuralol for bufuralol). The incubation mixtures were centrifuged at 13,000 × g for 5 min, and aliquots (5 μL) of the supernatants were analyzed by LC/MS/MS. For determination of *K*_i_ values for UGT1A1 and UGT1A9, human liver microsomes (0.2 mg/mL for 17β-estradiol; 0.1 mg/mL for propofol) were incubated with various concentrations of substrates (10−80 μM 17β-estradiol for UGT1A1 and 10–30 μM propofol for UGT1A9), 2 mM UDPGA, 25 μg/mL alamethicin, 10 mM MgCl_2_, and various concentrations of corydaline in 50 mM Tris buffer (pH 7.4) in a total incubation volume of 100 μL. Reactions were initiated by addition of UDPGA at 37 °C and stopped after 30 min by placement of the incubation tubes on ice and addition of 100 ng/mL ezetimibe (internal standard, 100 μL) in ice-cold methanol. The incubation mixtures were centrifuged at 13,000 × g for 5 min, followed by dilution of the supernatant (40 μL) with water (60 μL). Aliquots (5 μL) were analyzed by LC/MS/MS.

### 3.5. Data Analysis

IC_50_ values (concentration of inhibitor causing 50% inhibition of the original enzyme activity) were calculated using WinNonlin software, a non-linear regression analysis program (Pharsight, Mountain View, CA, USA). Apparent kinetic parameters for inhibitory potential (*K*_i_ values) of CYPs were estimated from the fitted curves using Enzyme Kinetics Ver. 1.3 program (Systat Software Inc., San Jose, CA, USA). For the enzyme kinetic analysis of UGT1A1 and UGT1A9 inhibition, the following mixed competitive-noncompetitive equation was fitted to the data points by nonlinear regression [30]:

$$V=\frac{V_{\max}\cdot \left[S\right]}{\left[S\right]\left(1+\frac{\left[I\right]}{\alpha \cdot K_{i}}\right)+K_{m}\left(1+\frac{\left[I\right]}{K_{i}}\right)}$$

Iterated variables were: [S]: substrate concentration; *V*: reaction velocity; [I]: inhibitor concentration; *V*_max_: the maximum reaction velocity; *K*_m_: Michaelis-Menten constant; *K*_i_: the inhibition constant; α: the mix of competitive-noncompetitive mechanisms.

## 4. Conclusions

In conclusion, an evaluation of the effects of corydaline on seven CYPs and four UGTs was conducted across a wide range of substrate and corydaline concentrations using *in vitro* human liver microsomes. CYP2C19 and CYP2C9 activity were potently inhibited by corydaline during incubation with NADPH in microsomes. Corydaline also inhibited CYP2D6-mediated bufuralol hydroxylation, CYP3A-mediated midazolam hydroxylation, UGT1A1-mediated 17β-estradiol 3-glucuronidation, and UGT1A9-mediated propofol glucuronidation in a dose-dependent manner. These results suggest that high uptake of corydaline from herbal medications may cause an interaction with drugs metabolized by CYP2C19 and CYP2C9 in some individuals. It is important to note, however, that the inhibition of CYP activities *in vitro* does not necessarily translate into drug interactions in clinical situations. Clinical trials to evaluate the inhibitory effects of corydaline on CYP2C19 and CYP2C9 remain to be conducted.

## Figures and Tables

**Table 1:** Effect of corydaline on CYP metabolic activity in pooled human liver microsomes (H161).

Enzymes	Marker reactions	IC_50_ (μM)		*K*_i_ (μM) (inhibition mode)
		no preincubation	with preincubation *	
1A2	Phenacetin *O*-deethylation	No inhibition **	No inhibition **	-
2A6	Coumarin 7-hydroxylation	No inhibition **	No inhibition **	-
2C8	Amodiaquine *N*-deethylation	No inhibition **	No inhibition **	-
2C9	Diclofenac 4-hydroxylation	26.2 ± 4.6	30.6 ± 4.1	7.1 ± 0.4 (noncompetitive)
2C19	*S*-Mephenytoin 4’-hydroxylation	11.7 ± 1.4	19.2 ± 1.8	1.7 ± 0.5 (competitive)
2D6	Bufuralol 1’-hydroxylation	64.5 ± 9.8	115.9 ± 7.6	27.3 ± 0.7 (competitive)
3A	Midazolam 1’-hydroxylation	>200	67.4 ± 9.8	-

* Corydaline was pre-incubated for 30 min in the presence of NADPH before addition of the substrate. ** There was no inhibition at 200 μM of corydaline. Cocktail substrate concentrations used for assessment of IC_50_ were as follows: 50 μM phenacetin, 2.5 μM coumarin, 2.5 μM amodiaquine, 10 μM diclofenac, 100 μM [*S*]-mephenytoin, 5.0 μM bufuralol, and 2.5 μM midazolam. The data represent mean ± standard deviation (*n* = 3).

**Table 2:** Effect of corydaline on UGT metabolic activity in pooled human liver microsomes (H161).

Enzymes	Marker reactions	IC_50_ (μM)	*K*_i_ (μM) (inhibition mode)
UGT1A1	17β-Estradiol 3-glucuronidation	137.1 ± 16.2	57.6 ± 13.0 (α = 0.555)
UGT1A4	Trifluoperazine *N*-glucuronidation	No inhibition *	-
UGT1A9	Propofol glucuronidation	39.4 ± 6.0	37.3 ± 14.4 (competitive)
UGT2B7	Azidothymidine glucuronidation	No inhibition *	-

* There was no inhibition at 1,000 μM of corydaline. The data represent mean ± standard deviation (*n* = 3). α: mix of competitive and noncompetitive mechanisms.

**Table 3:** LC/MS/MS measurement conditions for drug oxidation catalyzed by human CYP and drug glucuronidation catalyzed by human UGT.

Enzymes	Compound		SRM Transitions	Tube lens (V)	Collision energy (V)
CYP1A2	Metabolite	Acetaminophen	152.19 > 110.19	59	23
	Internal standard	^13^C_2_,^15^N-Acetaminophen	155.05 > 111.29	58	21
CYP2A6	Metabolite	7-Hydroxycoumarin	163.04 > 107.38	70	22
	Internal standard	d_5_-7-Hydroxycoumarin	168.00 > 112.53	73	22
CYP2C8	Metabolite	*N*-Desethylamodiaquine	328.01 > 282.64	45	19
	Internal standard	^13^C_2_,^15^N-Acetaminophen	155.05 > 111.29	58	21
CYP2C9	Metabolite	4-Hydroxydiclofenac	312.12 > 231.05	54	23
	Internal standard	^13^C_6_-4-Hydroxydiclofenac	318.49 > 237.28	54	20
CYP2C19	Metabolite	4-Hydroxymephenytoin	235.03 > 150.19	50	27
	Internal standard	d_3_-4-Hydroxymephenytoin	238.18 > 150.40	50	25
CYP2D6	Metabolite	1-Hydroxybufuralol	278.08 > 186.31	54	19
	Internal standard	d_9_-1-Hydroxybufuralol	287.12 > 187.09	54	20
CYP3A	Metabolite	1-Hydroxymidazolam	342.08 > 324.09	73	25
	Internal standard	d_3_-4-Hydroxymephenytoin	238.18 > 150.4	60	25
UGT1A1	Metabolite	17β-Estradiol 3-glucuronide	446.95 > 270.91	94	34
	Internal standard	Ezetimibe	408.07 > 271.43	45	21
UGT1A4	Product	Trifluoperazine *N*-glucuronide	584.20 > 408.13	94	27
	Internal standard	Meloxicam	352.05 > 115.38	63	20
UGT1A9	Metabolite	Propofol glucuronide	353.18 > 177.19	63	20
	Internal standard	Ezetimibe	408.07 > 271.43	45	21
UGT2B7	Metabolite	Azidothymidine glucuronide	444.26 > 127.02	70	22
	Internal standard	Meloxicam	352.05 > 115.38	63	20
